# High Expression of Ten Eleven Translocation 1 Is Associated with Poor Prognosis in Hepatocellular Carcinoma

**DOI:** 10.1155/2023/2664370

**Published:** 2023-05-04

**Authors:** Haopeng Wen, Tengfei Ji, Liteng Lin, Nan Cheng, Kangshun Zhu, Liangqi Cao

**Affiliations:** ^1^Department of Hepatobiliary Surgery, The Second Affiliated Hospital of Guangzhou Medical University, Guangzhou 510260, China; ^2^Department of Hepatobiliary Surgery, Affiliated Huadu Hospital, Southern Medical University (People's Hospital of Huadu District), Guangzhou 510000, China; ^3^Department of Minimally Invasive Interventional Radiology, The Second Affiliated Hospital of Guangzhou Medical University, Guangzhou 510260, China

## Abstract

**Background:**

DNA methylation patterns have been found to be distinct between tumor and normal patients. However, the effect of DNA demethylation enzymes, ten eleven translocation (TET) proteins, has not been comprehensively characterized in liver cancer. In this research, we sought to unravel the linkage of TET proteins with prognosis, immune characteristics and biological pathways in hepatocellular carcinoma (HCC).

**Materials and Methods:**

Four independent datasets with gene expression data and clinical data of HCC samples were downloaded from public databases. CIBERSORT, single sample Gene Set Enrichment Analysis (ssGSEA), MCP-counter, and TIMER were implemented to evaluate immune cell infiltration. limma was employed to screen differentially expressed genes (DEGs) between two groups. The demethylation-related risk model was established by using univariate Cox regression analysis, the least absolute shrinkage and selection operator (LASSO), and stepwise Akaike information criterion (stepAIC).

**Results:**

TET1 was significantly higher expressed in tumor samples than that in normal samples. HCC patients with advanced stages (III+IV) and grades (G3+G4) had higher TET1 expression compared to early stages (I+II) and grades (G1+G2). HCC samples with high TET1 expression had worse prognosis than that with low expression. High and low TET1 expression groups had distinct immune cell infiltration and response to immunotherapy and chemotherapy. We identified 90 DEGs related to DNA demethylation in high vs. low TET1 expression groups. Furthermore, we established a risk model based on 90 DEGs containing seven key prognostic genes (SERPINH1, CDC20, HACD2, SPHK1, UGT2B15, SLC1A5, and CYP2C9) with effectiveness and robustness in predicting HCC prognosis.

**Conclusions:**

Our study suggested TET1 as a potential indicator in HCC progression. TET1 was closely involved in immune infiltration and activation of oncogenic pathways. The DNA demethylation-related risk model was potential to be applied for predicting HCC prognosis in clinics.

## 1. Introduction

Liver cancer contributes to a proportion of 4.7% new cancer cases and 8.3% new cancer deaths worldwide according to the global cancer statistics in 2020 [1]. Hepatocellular carcinoma (HCC) is the most common histological type, comprising of approximately 75% of liver cancer patients [2]. The incidence of liver cancer in male populations are almost two times of that in female populations, as shown in 2020 cancer data [1]. Strikingly, liver cancer contributes to the second cancer death in male populations (10.5% of cancer deaths) [1]. Metastatic liver cancer patients have a poor overall survival, in spite of the treatment with molecular drugs, which results from the unavoidable drug resistance in most of the patients [3]. Also, due to the intratumor heterogeneity of liver cancer, the development of targeted therapies becomes even challenging [4]. Therefore, understanding the molecular mechanisms during liver cancer progression is of great importance for facilitating the exploration of novel therapeutic targets.

It is knowledgeable that the variations of tumor suppressor genes or protumor genes are the key inducers of cancer. In addition to solid genetic mutations, the alterations of epigenetic modifications are also a crucial factor in the onset process of cancer. Bulk of evidences have illustrated that DNA methylation profiles are distinct between normal and cancer genomes [5–7]. DNA methylation is under controlled by two classes of enzymes, methylation enzymes (DNMT3a and DNMT3b) [8] and demethylation enzymes (ten eleven translocation (TET) family) [9]. TET enzymes, consisting of consists of TET1, TET2, and TET3, are capable to reverse DNA methylation by oxidizing 5-methylcytosine (5mC) to 5-hydroxymethylcytosine (5hmC), 5-formylcytosine (5fC), and 5-carboxylcytosine (5caC) [10]. It has been shown that the aberrant expression and mutations of TET proteins are not rare in cancer patients [11, 12]. The expression levels of TET proteins are associated with tumor progression and metastasis, which offers a potential of TET proteins as markers in cancer prognosis and diagnosis [13, 14].

In this study, we focused on the effect of TET1 in HCC patients and sought to elucidate the potential crosstalk of TET1 with immune microenvironment in HCC. In addition, we identified key prognostic genes by extracting the TET1 and DNA methylation-related genes and established a risk model for predicting HCC prognosis. We demonstrated the potential of DNA methylation-related genes as prognostic markers in HCC patients.

## 2. Materials and Methods

### 2.1. Data Acquisition

TCGA-LIHC dataset (abbreviated as TCGA dataset) containing RNA sequencing (RNA-seq) data and clinical information was downloaded from Genomic Data Commons Data Portal (https://portal.gdc.cancer.gov/projects/TCGA-LIHC) through TCGA GDC API [15]. ICGC-LIRI-JP dataset (abbreviated as ICGC dataset) was downloaded from hepatocellular carcinoma database [16] (HCCDB, http://lifeome.net/database/hccdb/home.html). GSE14520 and GSE76427 datasets with microarray data were obtained from Gene Expression Omnibus (GEO) database [17] (https://www.ncbi.nlm.nih.gov/geo/query/acc.cgi?acc=GSE14520, https://www.ncbi.nlm.nih.gov/geo/query/acc.cgi?acc=GSE76427).

### 2.2. Data Preprocessing

For TCGA dataset, the HCC samples with survival time (over than 30 days and less than 10 years) and survival status were retained. Ensembl ID was transferred to gene symbol. The median value of gene expression was selected when the gene had multiple Ensembl IDs. After preprocessing, 334 HCC samples and 50 paracancerous (normal) samples were included in TCGA dataset. For two microarray datasets (GSE14520 and GSE76427), the probes were matched to the gene symbols according to the annotation file of microarray platform. The probes matching to multiple gene symbols were excluded, and the median expression level was used when there were multiple probes of one gene. A total of 221 and 115 HCC samples were included in GSE14520 and GSE76427 datasets, respectively. ICGC dataset included 212 HCC samples and 177 normal samples, and no preprocessing was performed for the ICGC data.

### 2.3. Immune Analysis

CIBERSORT, single sample gene set enrichment analysis (ssGSEA), Microenvironment Cell Populations-counter (MCP-counter), and Tumor IMmune Estimation Resource (TIMER) were employed to assess immune cell infiltration. CIBERSORT [18] (http://cibersort.stanford.edu/) is able to estimate the proportion of 22 immune cells from tumor mix based on a validated leukocyte gene signature matrix (LM22). MCP-counter [19] allows to detect the abundance of 10 cell populations including immune cell and stromal cell populations from the transcriptome of tumor tissues. SsGSEA [20] is a widely used methodology for evaluating the absolute enrichment score of a gene set for each sample. The gene sets of 28 immune cells were obtained from a previous study [21], and the ssGSEA scores of the immune cells were measured through GSVA R package [22]. TIMER [23] (http://timer.cistrome.org/) provides the interpretation of six major immune modules and visualizes the estimated proportion of tumor-infiltrated immune cells. TIDE [24] (http://tide.dfci.harvard.edu/) tool can predict the response to immune checkpoint inhibitors (ICIs) through estimating T cell status (exclusion and dysfunction) and infiltration of immunosuppressive cells including myeloid-derived suppressor cells (MDSCs), cancer-associated fibroblasts (CAFs), and M2 tumor-associated macrophages (TAMs).

### 2.4. Functional Enrichment Analysis of Biological Pathways

Gene set enrichment analysis (GSEA) software [25] was applied to identify enriched pathways with an ordered gene set. Kyoto Encyclopedia of Genes and Genomes (KEGG) pathways “c2.cp.kegg.v7.5.1.symbols.gmt” and hallmark pathways “h.all.v7.5.1.symbols.gmt” were downloaded from Molecular Signature Database (MSigDB, https://www.gsea-msigdb.org/gsea/msigdb/).

### 2.5. Differential Analysis

Differentially expressed genes (DEGs) between two groups were identified by limma R package [26] based on their gene expression profiles. False discovery rate (FDR) < 0.05 and |log2 fold change (*FC*)| > 1 were set as thresholds to screen significant DEGs. ClusterProfiler R package [27] was implemented to annotate the significantly enriched Gene Ontology (GO) terms and KEGG pathways of DEGs.

### 2.6. Construction of a Risk Model for Predicting HCC Prognosis

The gene sets of two DNA demethylation-related biological processes (BPs) GOBP_DNA_METHYLATION_OR_DEMETHYLATION and GOBP_POSITIVE_REGULATION_OF_DNA_DEMETHYLATION were downloaded from MSigDB. The enrichment score of the two BPs was calculated by ssGSEA via GSVA R package. Pearson correlation analysis was performed between DEGs and TET1 and DEGs and the ssGSEA score of BPs by using Hmisc R package (https://cran.r-project.org/web/packages/Hmisc/index.html). The DEGs with significant correlations both with TET1 and BPs were screened under |*R*| > 0.2 and *P* < 0.05. Next, the DEGs were further screened by univariate Cox regression analysis, least absolute shrinkage and selection operator (LASSO) [28], and stepwise Akaike information criterion (stepAIC) [29]. Finally, the risk model was constructed with gene expression and Lasso coefficients. Risk score = *Σ*(Exp i∗beta i). Exp indicates the expression levels of genes (*i*), and beta indicates the LASSO coefficients of corresponding genes. The effectiveness and efficiency of the risk model were validated by Kaplan-Meier survival analysis and receiver operating characteristic (ROC) curve analysis.

### 2.7. Statistical Analysis

The statistical analysis used in this study was performed in R software (v4.2.0). Wilcoxon test was used to measure the difference between two groups. ANOVA test was conducted to detect the difference among four groups. *P* < 0.05 was determined as statistically significant. The visualization of analyzed results was supported by the Sangerbox platform [30] (http://sangerbox.com/).

## 3. Results

### 3.1. TET1 Expression Was Correlated with the Prognosis and Clinical Characteristics in HCC

**Figure 1:**
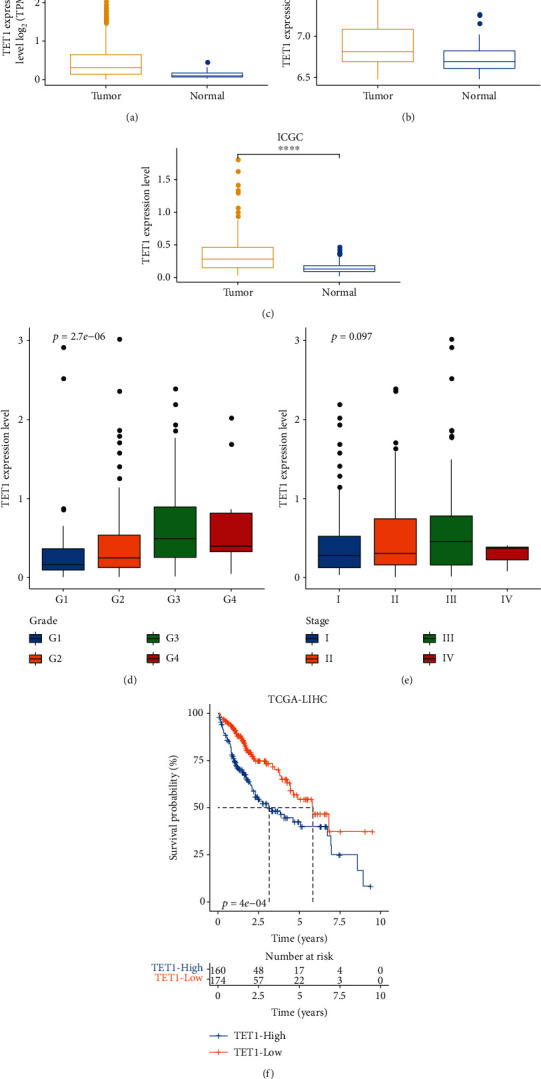
TET1 expression was associated with HCC progression and survival. (a–c) The expression level of TET1 in normal and tumor samples in three datasets. Wilcoxon test was performed. (d, e) The expression level of TET1 in different grades and stages in TCGA dataset. ANOVA test was conducted. (f) Kaplan-Meier survival analysis of TET1-high and TET1-low groups in TCGA dataset. Log-rank test was performed. ^∗∗∗∗^*P* < 0.0001.

**Figure 2:**
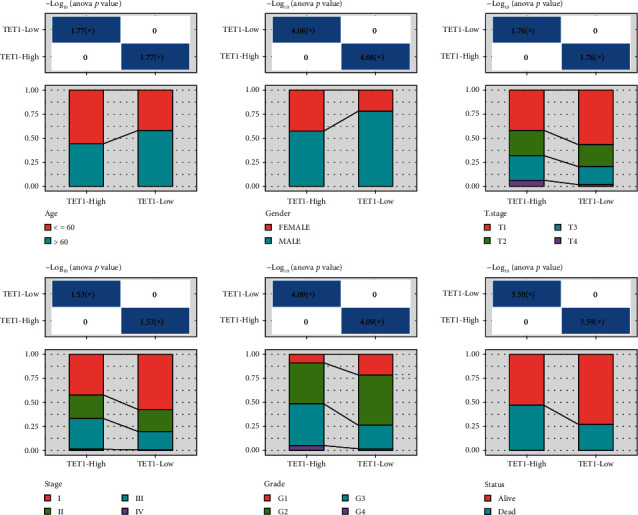
TET1-high and TET1-low groups had different distribution of clinical characteristics including age, gender, T stage, stages I-IV, grade, and survival status in TCGA dataset.

To evaluate the TET alteration in HCC, we assessed the expression levels of TET1, TET2, and TET3 in three independent datasets (TCGA, GSE76427, and ICGC). As a result, only TET1 was differently expressed between tumor and normal samples in TCGA, GSE76427, and ICGC datasets (*P* < 0.0001, Figures 1(a)–1(c)). An upregulated expression level of TET1 was observed in HCC samples compared with normal samples. In addition, the samples with late grades or stages showed higher TET1 expression (Figures 1(d) and 1(e)), suggesting that high expression of TET1 may be a risk factor of HCC progression. To examine the performance of TET1 as a prognostic biomarker in HCC, we divided HCC samples into high TET1 expression (TET1-high) and low TET expression (TET1-low) groups according to the median value. Not surprisingly, samples in TET1-low group had obviously longer overall survival than that in TET1-low group (*P* = 4*e* − 04, Figure 1(f)). Moreover, the distribution of clinical characteristics showed significant differences between TET1-low and TET1-high groups (Figure 2). The proportion of samples with early stages (T1, stage I, and G1) was higher in TET1-low group than that in TET1-high group. Conversely, TET1-high group had a higher proportion of late stages than TET1-low group. In accordant with the above observations, dead samples were more accumulated in TET1-high group compared with TET1-low group. The significant difference of clinical characteristics and prognosis in two TET1 groups indicated that TET1 was importantly involved in HCC progression.

### 3.2. Immune Characteristics and Biological Analysis in Two TET1 Groups

**Figure 3:**
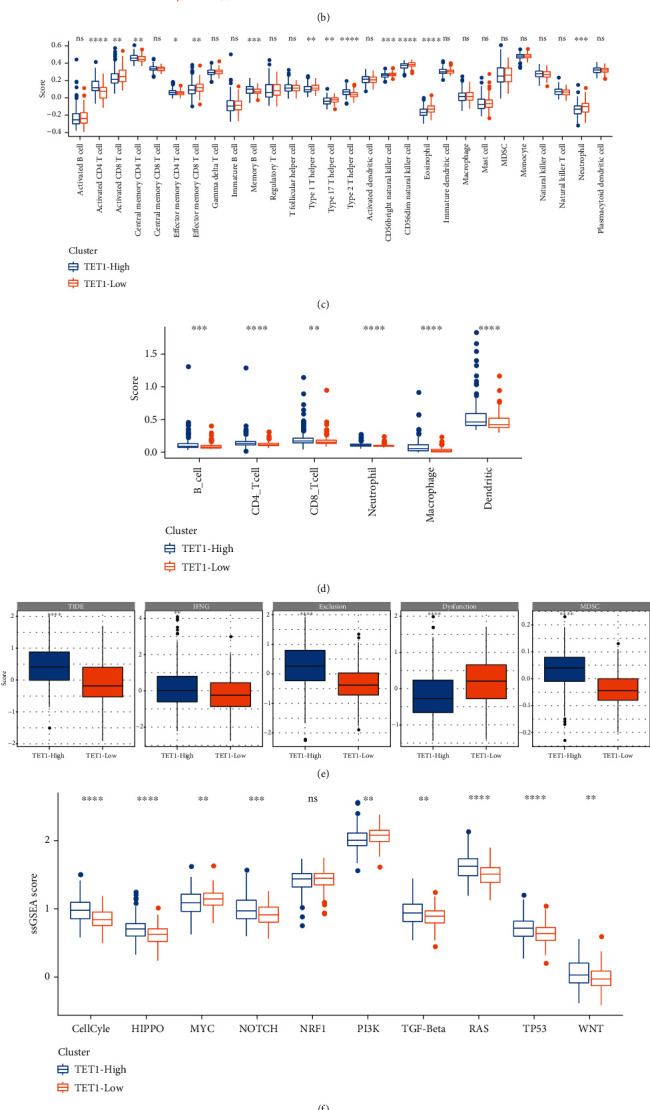
Immune characteristics and biological pathways in TET1-high and TET-low groups in TCGA dataset. (a–d) The estimated enrichment of immune cells analyzed by CIBERSORT, MCP-counter, ssGSEA, and TIMER. (e) TIDE analysis on TET1-high and TET-low groups. (f) The ssGSEA score of 10 oncogenic pathways. (g) GSEA on TET1-high vs. TET1-low groups. Wilcoxon test was conducted. ns: not significant. ^∗^*P* < 0.05; ^∗∗^*P* < 0.01; ^∗∗∗^*P* < 0.001; ^∗∗∗∗^*P* < 0.0001.

Evidence has shown that TET proteins play a regulatory role in immune cell development and orchestrate cell differentiation in tumorigenesis [31]. We compared the immune cell infiltration in two TET1 groups through multiple strategies including CIBERSORT, ssGSEA, MCP-counter, and TIMER. The results presented that multiple types of immune cells were differently enriched in TET1-high and TET1-low groups, such as macrophages, CD8 T cells, and natural killer (NK) cells (Figures 3(a)–3(d)). In the response to immunotherapy, TET1-high group showed higher TIDE score than TET1-low group, which suggested higher immune evasion of TET1-high group possibly resulting from T cell exclusion and infiltration of MDSC (Figure 3(e)). Furthermore, we assessed 10 oncogenic pathways in two TET1 groups and found that 9 oncogenic pathways had distinct enrichment scores between two groups (*P* < 0.01, Figure 3(f)). TET1-high group had higher enrichment score of most oncogenic pathways such as Hippo, Notch, TGF-beta, cell cycle, TP53, and Wnt signaling pathways than TET1-low group. GSEA results showed that metabolic pathways fatty acid metabolism and retinol metabolism were more activated in TET1-low group compared with TET-high group (Figure 3(g)).

**Figure 4:**
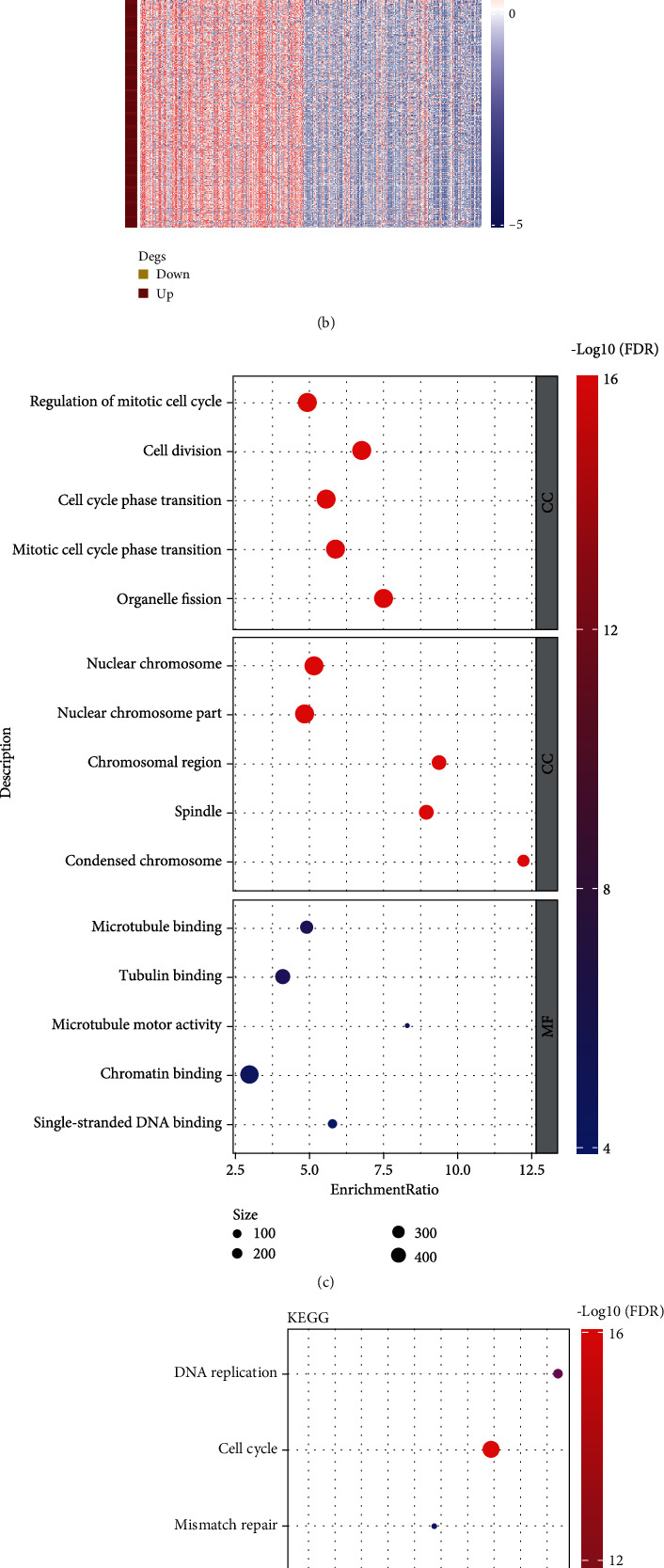
Differential analysis between TET1-high and TET1-low groups in TCGA dataset. (a) Volcano plot of DEGs between TET1-high and TET1-low groups. (B) Heat map of the expression of DEGs. (c) GO enrichment analysis showed the top five enriched biological pathways (BP), cellular components (CC), and molecular function (MF). (D) KEGG pathway analysis showed the top 10 enriched pathways. FDR: false discovery rate. BP: biological process. CC: cellular component. MF: molecular function.

To further explore the difference of activated biological pathways in two TET1 groups, we performed differential analysis and identified a total of 516 DEGs between two groups. We identified 404 upregulated DEGs and 112 downregulated DEGs in TET1-high group (Figures 4(a) and 4(b)). Functional analysis on the upregulated DEGs revealed that cell cycle and DNA repair-related pathways were strikingly enriched (Figure 4(c)). The above results suggested that TET1 may serve as an important role in immune cell orchestration and tumorigenesis.

### 3.3. Construction and Verification of a Risk Model Related to TET1 and Demethylation-Related Genes

**Figure 5:**
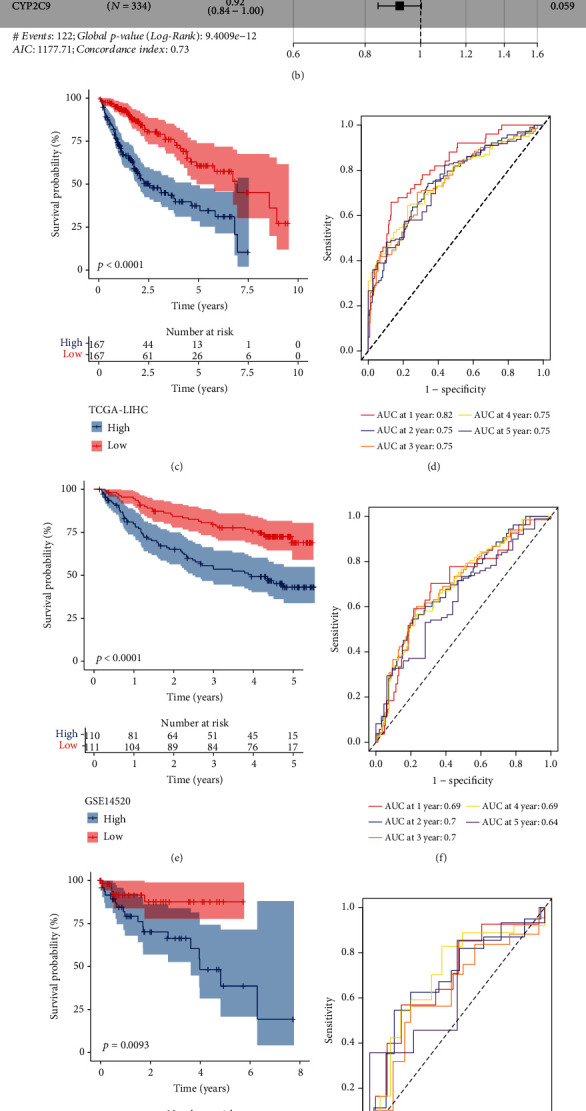
Construction and verification of TET1 and demethylation-related risk model. (a) Venn plot of TET1-related DEGs and demethylation-related DEGs. (b) Hazard ratio of the 7 prognostic genes determined by stepAIC. Kaplan-Meier survival curves of high-risk and low-risk groups in TCGA (c), GSE14520 (e), GSE76427 (g), and ICGC (i) datasets. ROC curves of the risk model in predicting 1- to 5-year survival in TCGA (d), GSE14520 (f), GSE76427 (h), and ICGC (j) datasets.

TET1 proteins serve an important role in DNA demethylation. Therefore, we tried to obtain the DEGs associated with both TET1 and DNA demethylation. To reach this goal, we accessed DNA demethylation-related BPs from MsigDB database (GOBP_DNA_METHYLATION_OR_DEMETHYLATION and GOBP_POSITIVE_REGULATION_OF_DNA_DEMETHYLATION). Correlation analysis was conducted between DEGs and TET1 or the two demethylation-related BPs, and a total of 90 DEGs were screened to be significantly correlated with both TET1 and the ssGSEA score of two BPs (|*R*| > 0.2; *P* < 0.05, Figure 5(a)). The 90 DEGs were used as a basis for constructing a risk model. Subsequently, we performed a series of methodologies to screen key DEGs for reaching the optimal model. First of all, univariate Cox regression analysis identified the genes significantly associated with overall survival (defined as prognostic genes) in TCGA dataset. Then, the number of prognostic genes were compressed by LASSO and stepAIC. LASSO analysis identified 10 prognostic genes when the lambda and the model reached the optimal (lambda = 0.0294) (Figure S1). Lastly, stepAIC confirmed the 7 prognostic genes as the final genes for constructing the risk model defined as follows (Figure 5(b)):

(1) $$\begin{array}{c} \text{Risk score}=-0.351*\text{SERPINH}1+0.271*\text{CDC}20+0.313*\text{HACD}2-0.149*\text{SPHK}1-0.089*\text{UGT}2\text{B}15+0.324*\text{SLC}1\text{A}5-0.084*\text{CYP}2\text{C}9. \end{array}$$

The risk model was verified in four independent datasets. Each sample obtained a risk score and two groups (high-risk and low-risk groups) were determined according to the median value of risk score. Kaplan-Meier survival analysis carried out significant differences on the overall survival between two risk groups in four independent datasets (*P* < 0.05, Figures 5(c), 5(e), 5(g), and 5(i)). Moreover, ROC curve analysis verified that the risk score was efficient to predict 1- to 5-year survival (Figures 5(d), 5(f), 5(h), and 5(j)), indicating the risk model was effective and reliable in predicting prognosis for HCC patients.

### 3.4. The Linkage of Risk Score with Clinical Characteristics, Immune Characteristics and Biological Pathways

**Figure 6:**
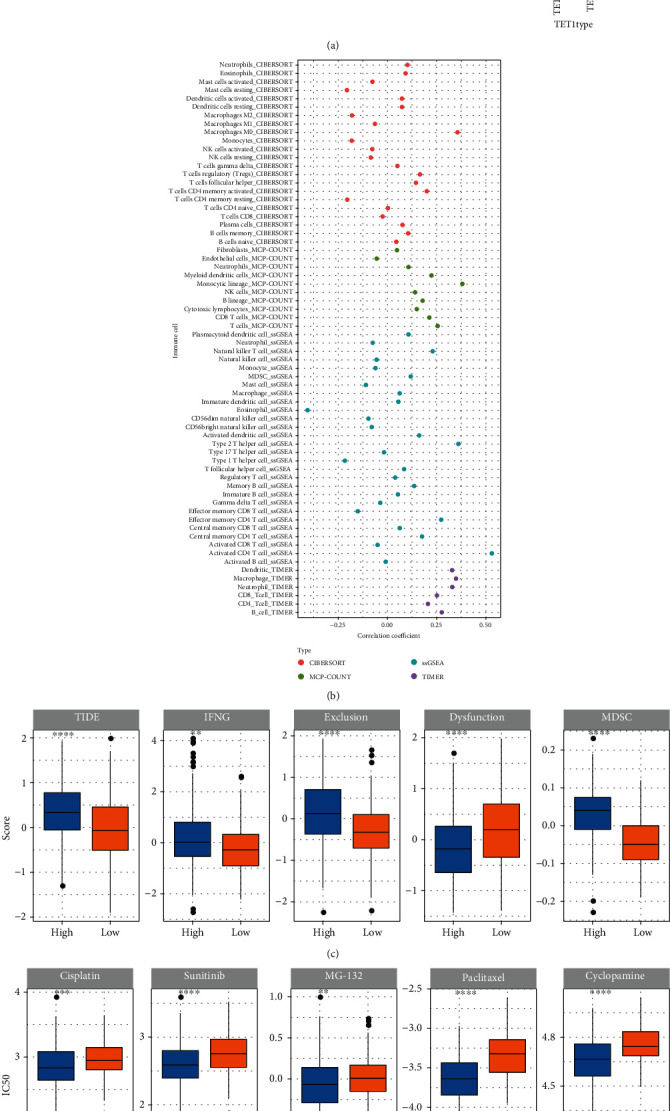
The relation of risk score with clinical characteristics, immune characteristics, and chemotherapeutic drugs. (a) The risk score in different genders, ages, stages, grades, and TET1 groups. (b) Correlation analysis between risk score and immune cell infiltration. (c) TIDE analysis of two risk groups. (d) The estimated IC50 of chemotherapeutic drugs in two risk groups. Wilcoxon test was performed between two groups, and ANOVA was performed among four groups. ns: not significant. ^∗^*P* < 0.05; ^∗∗^*P* < 0.01; ^∗∗∗^*P* < 0.001; ^∗∗∗∗^*P* < 0.0001.

In the relation between risk score and clinical characteristics, we observed that there were evident differences on the risk score between different genders, stages, and grades. Strikingly, the risk score increased with the advancing stages and grades (Figure 6(a)). We also analyzed the association of TET1 with the risk score, and the result shown that TET1-high group had markedly higher risk score than TET1-low group (*P* < 0.0001, Figure 6(a)), implying that the 7 prognostic genes in the risk model may be involved in the regulation of TET1.

We investigated the immune microenvironment of two risk groups by CIBERSORT, MCP-counter, ssGSEA, and TIMER. Some immune cells were differently enriched between two risk groups, such as macrophages, memory CD4 T cells, and dendritic cells (Figure S2). Notably, significant correlations were observed between risk score and M0 macrophages, type 2 helper T cells, monocytic lineage, and activated CD4 T cells (Figure 6(b)). TIDE analysis predicted that high-risk group was easier to escape from immunotherapy due to its high T cell exclusion and high infiltration of MDSCs (Figure 6(c)). However, high-risk group may benefit more from chemotherapeutic drugs than low-risk groups, because the estimated IC50 of cisplatin, sunitinib, MG-132, paclitaxel, and cyclopamine were lower in high-risk group (Figure 6(d)).

**Figure 7:**
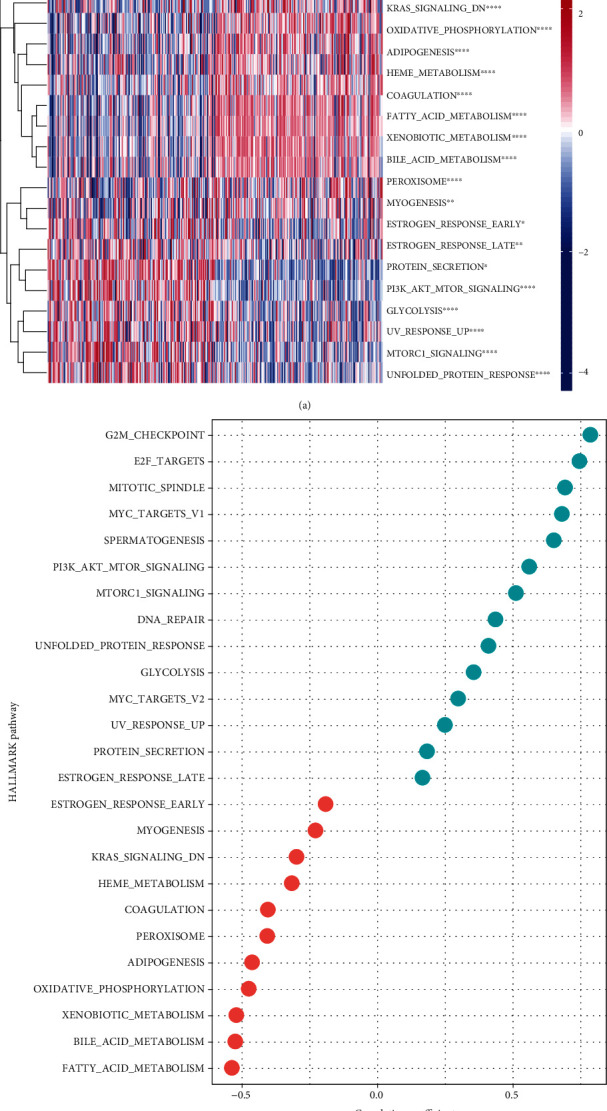
Pathway analysis of two risk groups. (a) Heat map of differently enriched pathways in two risk groups. Wilcoxon test was performed. Red and blue indicate relatively activated and suppressed, respectively. (b) Correlation analysis between risk score and hallmark pathways. Red and green indicate negative and positive correlations, respectively.

To explore whether two risk groups had different biological activities, we included all hallmark pathways downloaded from MSigDB and calculated the ssGSEA score for each pathway in TCGA dataset. By comparing the ssGSEA score in two risk groups, we identified a total of 25 pathways markedly differently activated between two groups (Figure 7(a)). Clustering results of these pathways presented that two risk groups had distinct patterns of activated pathways. Cell cycle-related pathways such as E2F targets, MYC target V1, MYC target V2, G2M checkpoint, and DNA repair were evidently activated in high-risk group, while metabolism-related pathways were significantly activated in low-risk group such as adipogenesis, fatty acid metabolism, heme metabolism, xenobiotic metabolism, and bile acid metabolism. In addition, glycolysis, PI3K Akt mTOR signaling, and unfolded protein response were also found to be more enriched in high-risk group. In the relation of risk score with the above pathways, consistent results were outputted that a positive correlation was shown between cell cycle-related pathways and risk score, while a negative correlation was shown between metabolic pathways and risk score (Figure 7(b)).

### 3.5. Optimizing the Clinical Application of Risk Score

**Figure 8:**
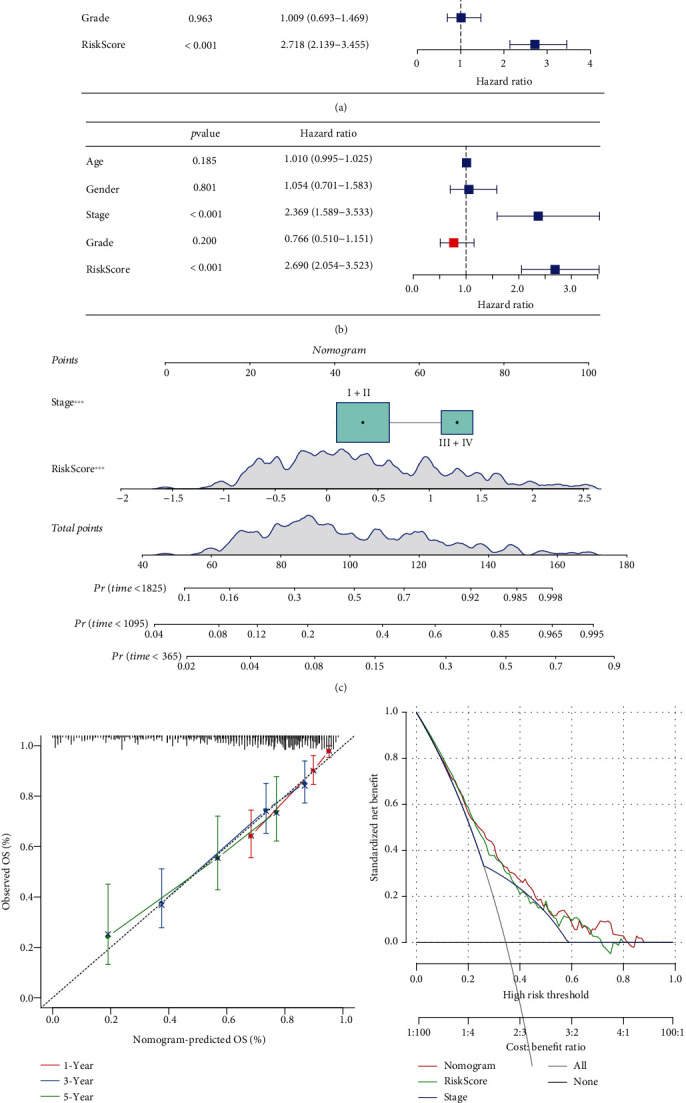
Construction of a nomogram based on risk score and clinical characteristics. (a) Univariate Cox regression analysis of risk score and clinical characteristics. (b) Multivariate Cox regression analysis of risk score and clinical characteristics. (c) The nomogram based on risk score and stage. (d) Calibration curve of the predicted OS and the observed OS. (e) DCA curve of risk score, nomogram, and stage. OS: overall survival. ^∗∗∗^*P* < 0.001.

To make the risk score more conveniently used in clinical situations, we introduced a nomogram system involving all prognostic factors. Cox regression was applied to determine the variables involved in the nomogram. As a result, only stage and risk score were independent risk factors with hazard ratio (HR) of 2.369 and 2.690, respectively, in multivariate regression (Figures 8(a) and 8(b)). Therefore, stage and risk score were used to construct the nomogram for predicting the 1-year, 3-year, and 5-year survivals (Figure 8(c)). The predicted 1-year, 3-year, and 5-year overall survivals by the nomogram were almost overlapped with the actual ones (Figure 8(d)), indicating that the nomogram was reliable. Moreover, decision curve analysis (DCA) demonstrated that the nomogram had the best net benefit that the patients could obtain from (Figure 8(e)).

## 4. Discussion

Previous studies have discovered that the aberrant DNA methylation patterns with global hypomethylation are associated with cancer progression in HCC [32, 33]. In the demethylation process, TET proteins are responsible for the removal of methylation and the alteration of DNA methylation patterns. To further understand the role of TET proteins and DNA demethylation-related genes in HCC, this study characterized the linkage of demethylation with survival, clinical characteristics, tumor microenvironment (TME), and biological pathways using various strategies of bioinformatics analysis. We emphasized the importance of TET proteins in HCC progression and the response to clinical treatment.

First of all, we compared the expression levels of TET proteins between tumor and normal samples and found that only TET1 had an elevated expression level in HCC samples compared with the normal samples in three independent datasets. The HCC samples with late grades (G2-G4) and stages (II-IV) had higher TET1 expression than that with the early grade (G1) and stage (I), suggesting a linkage between TET1 expression and prognosis. To demonstrate the speculation, we stratified HCC samples into two groups by the median cut-off of TET1 expression. Not surprisingly, TET1-high group showed evidently shorter overall survival than TET1-low group. High expression level of TET1 may lead to high activity of demethylation process, which supported that the downregulated methylations were associated with poor prognosis.

TME is a critical component affecting cancer invasion, metastasis, and even the efficiency of immunotherapy and chemotherapy [34]. To understand the potential effect of TET1 in TME, we assessed the relation of two TET1 expression groups with immune infiltration through multiple methodologies. As a result, some immune cells were found to be differently enriched in two TET1 groups, indicated that DNA methylation may function an effect in TME regulation. Lines of studies have found that DNA methylation patterns had an influence in immune characteristics in various cancer types. For example, Mitra et al. identified three immune methylation-based clusters showing different immune cell infiltration and prognosis in metastatic melanoma [35]. Meng et al. delineated a landscape of DNA methylation regulators in gastric cancer and found the extensive dysregulation of the regulators [36]. Moreover, the expression of DNA methylation regulators was closely related to immune cell infiltration, where TET1 expression was related to the enrichment of activated dendritic cells, neutrophils, and type 17 T helper cells [36]. In our results, TET1-high and TET1-low groups showed different enrichment of multiple immune cells such as macrophages, natural killer cells, and neutrophils. It could be implied that TET1 was involved in the crosstalk with immune microenvironment. Strikingly, TET1-high group was easier to escape from immunotherapy than TET1-low group, suggesting that TET1 had a potential to serve as an indicator for guiding immunotherapy in HCC.

To reveal the interplay of TET1 with biological pathways, we assessed oncogenic pathway and KEGG pathway in TET1-high and TET1-low groups. Of 10 oncogenic pathways, it was remarkable that 9 of them were differentially enriched in two TET1 groups, supporting that TET1 expression was related to the activation of oncogenic pathways. Specifically, TET1-high group had significantly higher enrichment of most of oncogenic pathways such as cell cycle, Hippo, Notch, TGF-*β*, TP53, and Wnt signaling pathways. Evidence has shown that there is a substantial difference on DNA methylation of oncogenic pathways such as Hippo and Wnt between HCC and normal samples [37]. We speculated that the demethylation effect resulting from high TET1 expression activated the expression of genes involved in the oncogenic pathways. In addition, KEGG enrichment analysis on the DEGs between two TET1 groups unveiled that cell cycle-related and DNA repair-related pathways were more activated in the upregulated DEGs of TET1-high group. The results further sustained the important role of TET1 in regulating oncogenic pathways.

Due to the close relation of TET1 with survival and oncogenic pathways, we dug out a bulk of genes related to TET1 and DNA demethylation process for constructing a risk model. Based on TET1 and DNA demethylation-related genes, we identified seven key prognostic genes including SERPINH1, CDC20, HACD2, SPHK1, UGT2B15, SLC1A5, and CYP2C9 for the model construction. The risk model manifested superior prediction efficiency of HCC prognosis in four independent datasets. Notably, TET1-high group had extraordinarily higher risk score than TET1-low group. The risk score increased with the advancing stages and grades, which was consistent with the observation regarding TET1 expression. Therefore, the seven key prognostic genes may be closely involved in the TET1-mediated demethylation. Increased expression of CDC20 was reported to be associated with HCC progression through promoting cell proliferation and inhibiting apoptosis [38, 39]. SPHK1 was found to be upregulated in HCC and could induce epithelial-mesenchymal transition (EMT) process [40]. Few studies have reported the other five prognostic genes on their molecular mechanisms in HCC.

However, our study only relied on the bioinformatics analysis, the mechanism of TET1 in HCC development and progression needed verification in molecular experiments. We did not simultaneously compare the DNA methylation patterns relating TET1 expression. In addition, the seven-gene risk model should be further validated in clinical samples, and the potential mechanisms of the seven key prognostic genes in TET1-mediated demethylation needed to be clarified in future study.

## 5. Conclusions

In conclusion, our study confirmed the overexpression of TET1 in HCC patients and unveiled the relation of TET1 expression with survival, clinical stages, immune cell infiltration, the response to immunotherapy and chemotherapy, and oncogenic pathways. We identified seven key prognostic genes related to TET1 and DNA demethylation and established a nomogram for effectively predicting HCC prognosis.

## Data Availability

The data used to support the findings of this study are included within the article.

## Supplementary Materials

Supplementary 1 Figure S1: LASSO regression of the prognostic genes. (A) The coefficients of prognostic genes with the changing lambda values. Red dotted line indicates lambda = 0.0294. (B) Partial likelihood deviance of different lambda values. Red dot indicates lambda = 0.0294.

Supplementary 2 Figure S2: the enrichment score of immune cells analyzed by CIBERSORT (A), MCP-counter (B), ssGSEA (C), and TIMER (D). The Wilcoxon test was conducted. ns: not significant. ^∗^*P* < 0.05; ^∗∗^*P* < 0.01; ^∗∗∗^*P* < 0.001; ^∗∗∗∗^*P* < 0.0001.
