# Supplementary figures and images for: High Expression of Ten Eleven Translocation 1 Is Associated with Poor Prognosis in Hepatocellular Carcinoma
Source: Mediators Inflamm. 2023 May 4;2023:2664370. doi: 10.1155/2023/2664370 (PMC10175022; doi:10.1155/2023/2664370)

**A**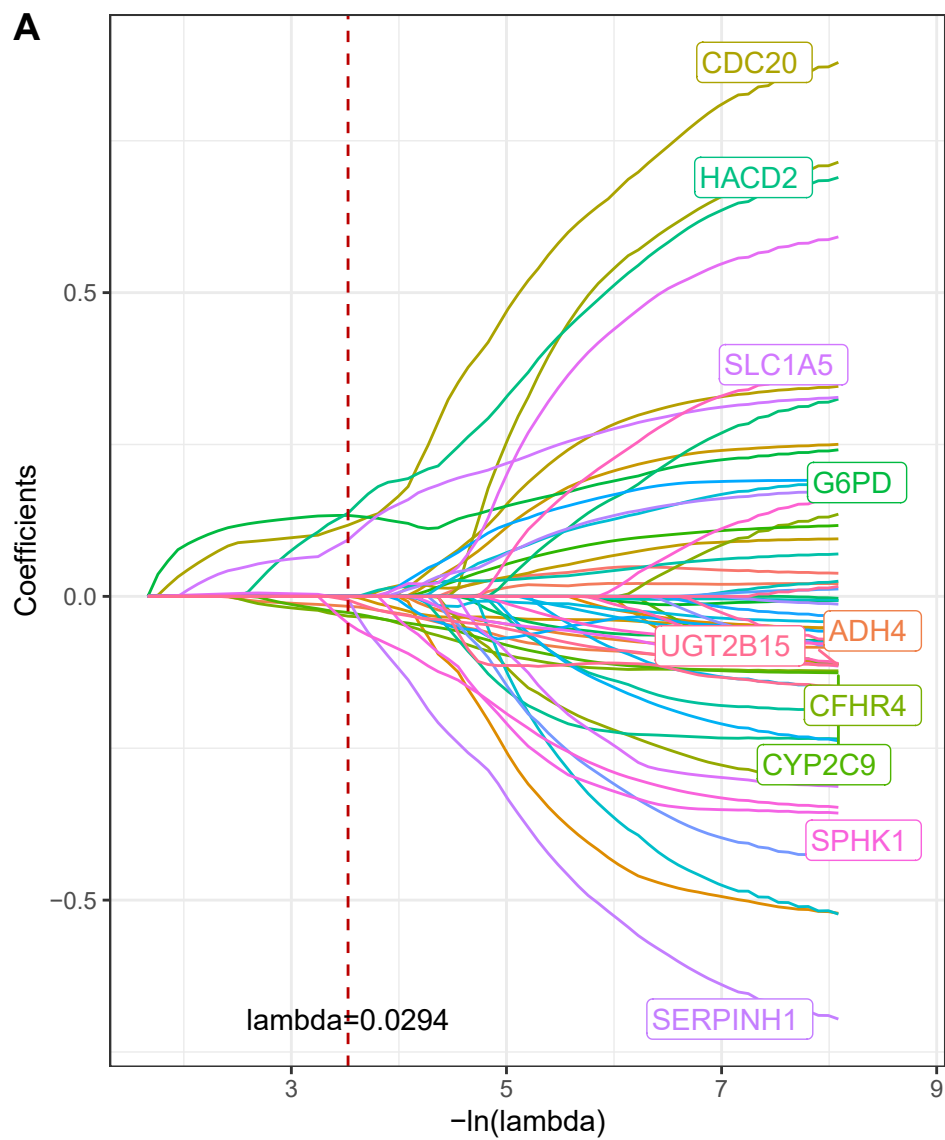**B**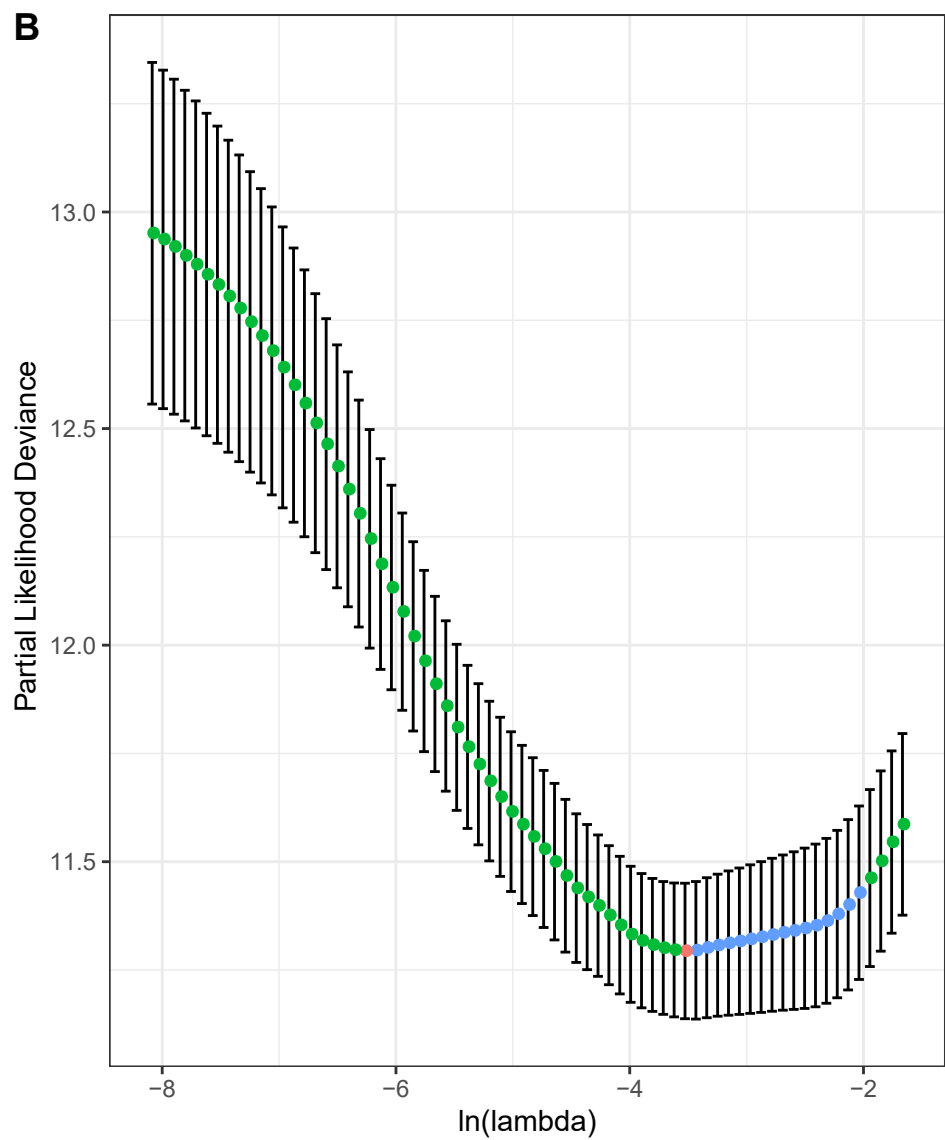

Supplement: Supplementary 1 — Figure S1: LASSO regression of the prognostic genes. (A) The coefficients of prognostic genes with the changing lambda values. Red dotted line indicates lambda = 0.0294. (B) Partial likelihood deviance of different lambda values. Red dot indicates lambda = 0.0294. [file 2664370.f1.pdf]

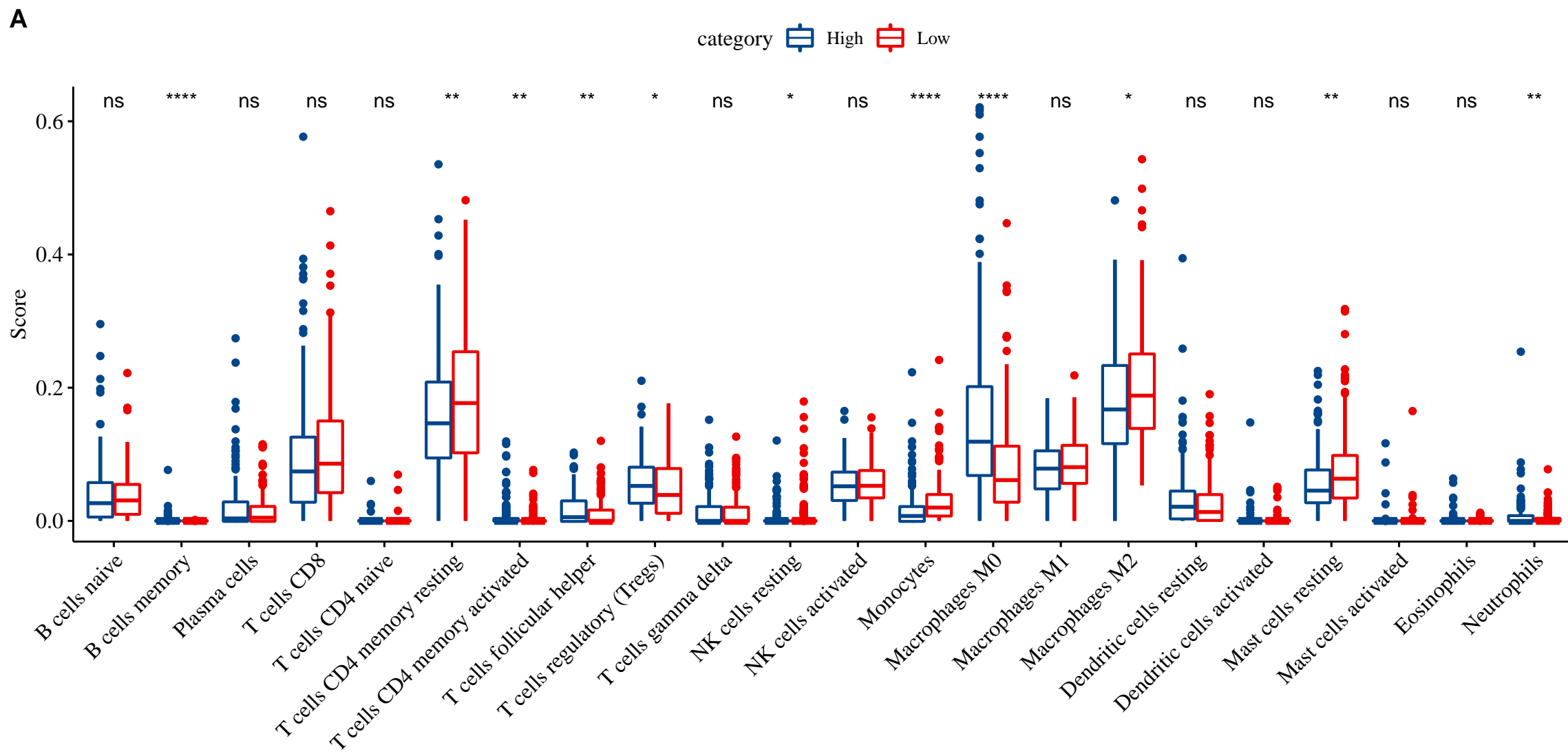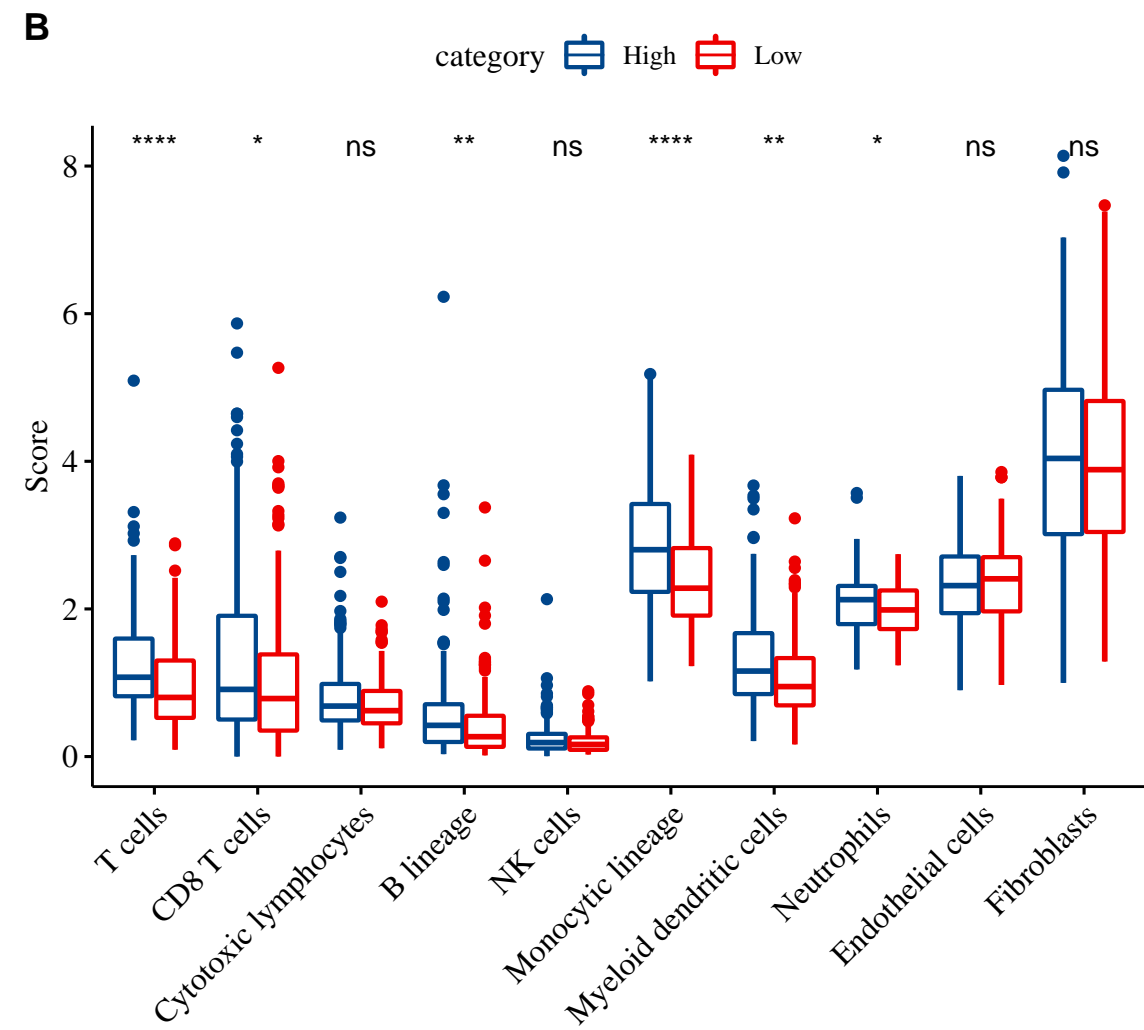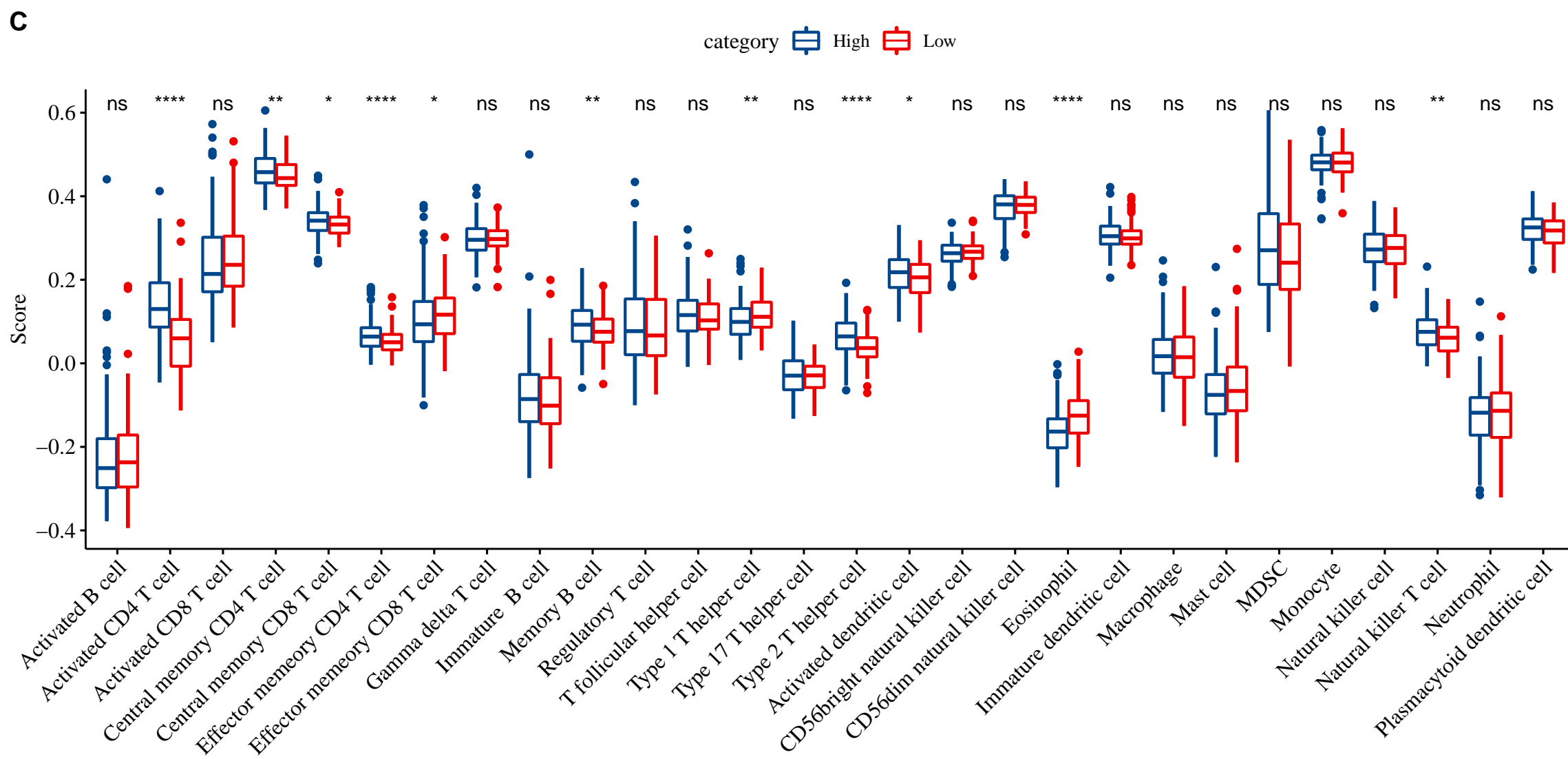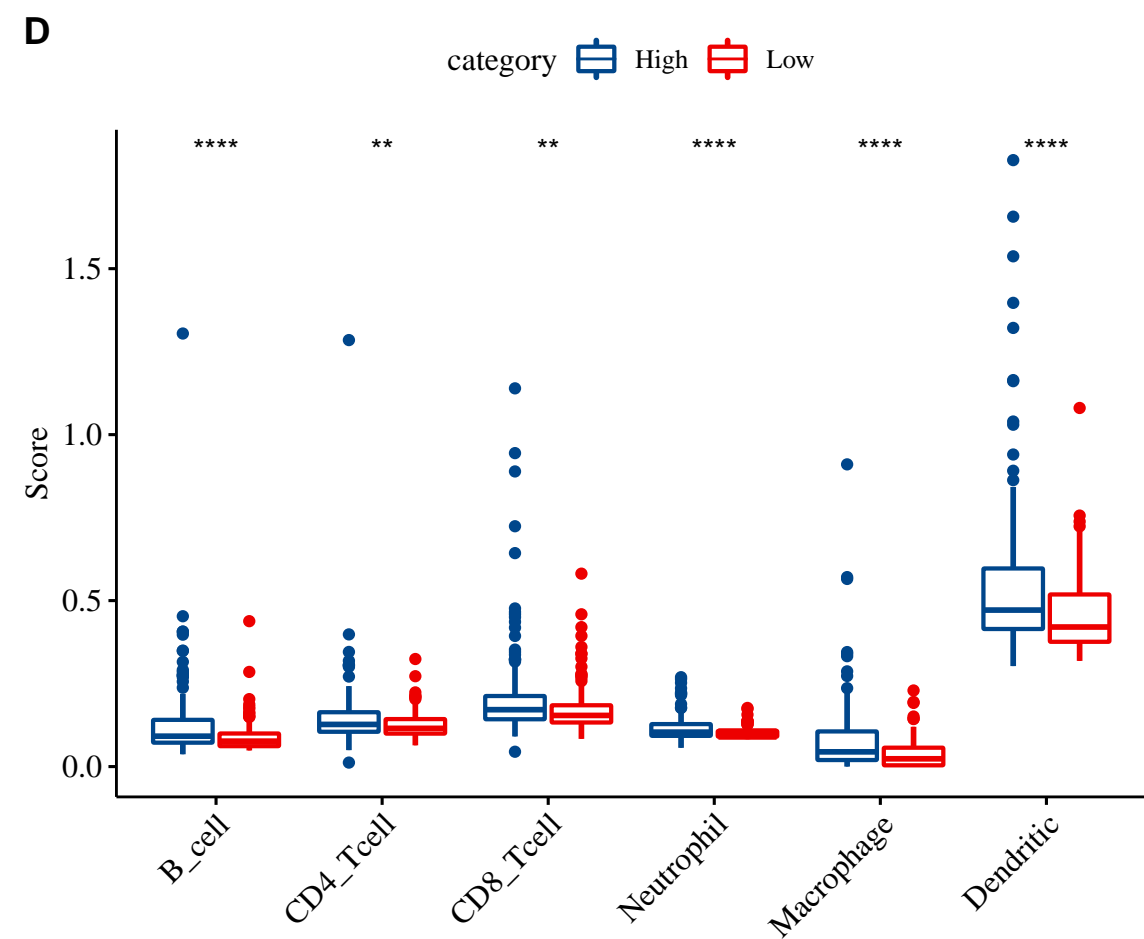

Supplement: Supplementary 2 — Figure S2: the enrichment score of immune cells analyzed by CIBERSORT (A), MCP-counter (B), ssGSEA (C), and TIMER (D). The Wilcoxon test was conducted. ns: not significant. ∗P < 0.05; ∗∗P < 0.01; ∗∗∗P < 0.001; ∗∗∗∗P < 0.0001. [file 2664370.f2.pdf]
